# Perturbation of the Developmental Potential of Preimplantation Mouse Embryos by Hydroxyurea

**DOI:** 10.3390/ijerph7052033

**Published:** 2010-04-28

**Authors:** Mariam Sampson, Anthony E. Archibong, Adriane Powell, Brandon Strange, Shannon Roberson, Edward R. Hills, Phillip Bourne

**Affiliations:** Department of Obstetrics and Gynecology, Meharry Medical College, Nashville, TN 37208-3599, USA; E-Mails: msampson@mmc.edu (M.S.); apowell@mmc.edu (A.P.); bstrange@mmc.edu (B.S.); sroberson@mmc.edu (S.R.); ehills@mmc.edu (E.H.); pbourne@mmc.edu (P.B.)

**Keywords:** ovarian weight, estradiol-17β ovulation rate, 2-cell embryo, blastocyst, hydroxyurea

## Abstract

Women are advised not to attempt pregnancy while on hydroxyurea (HU) due to the teratogenic effects of this agent, based on results obtained from animal studies. Several case reports suggest that HU may have minimal or no teratogenic effects on the developing human fetus. Fourteen cases of HU therapy in pregnant patients diagnosed with acute or chronic myelogenous leukemia, primary thrombocythemia, or sickle cell disease (SCD) have been reported. Three pregnancies were terminated by elective abortion; 1 woman developed eclampsia and delivered a phenotypically normal stillborn infant. All other patients delivered live, healthy infants without congenital anomalies. We contend that case studies such as these have too few patients and cannot effectively address the adverse effect of HU on preimplantation embryo or fetuses. The objective of this study was to assess the risks associated with a clinically relevant dose of HU used for the treatment of SCD, on ovulation rate and embryo development, using adult C57BL/6J female mice as a model. In Experiment 1, adult female mice were randomly assigned to a treatment or a control group (N = 20/group). Treatment consisted of oral HU (30 mg/kg) for 28 days; while control mice received saline (HU vehicle). Five days to the cessation of HU dosing, all mice were subjected to folliculogenesis induction with pregnant mare serum gonadotropin (PMSG). Five mice/group were anesthetized at 48 hours post PMSG to facilitate blood collection via cardiac puncture for estradiol-17β (E_2_) measurement by RIA. Ovulation was induced in the remaining mice at 48 hours post PMSG with human chorionic gonadotropin (hCG) and immediately caged with adult males for mating. Five plugged female mice/group were sacrificed for the determination of ovulation rate. The remaining mated mice were sacrificed about 26 hours post hCG, ovaries excised and weighed and embryos harvested and cultured in Whitten’s medium (WM) supplemented with CZBt. In Experiments 2 and 3, (N = 10/Experiment) folliculogenesis and ovulation were induced in untreated mice followed by mating. Recovered embryos were either exposed continuously (Experiment 2) or intermittently (Experiment 3) to bioavailable HU (18 μg HU/mL of WM + CZBt) or WM + CZBt only (control). Treated mice sustained decreased ovarian wt, ovulation rate and circulating E_2_ compared with controls (P < 0.05). Fewer embryos retrieved from HU-treated mice developed to blastocyst stage (32%) compared with those from controls (60%; P < 0.05). Furthermore, continuous or intermittent *in vitro* exposures of embryos to HU also resulted in reduced development to blastocyst stage (continuous HU, 9 *vs.* control, 63%; P < 0.05; intermittent HU, 20 *vs.* control, 62%; P < 0.05) with embryos exposed continuously to HU *in vitro* fairing worse. Even though HU is well tolerated, our data suggest that it compromises folliculogenesis and the ability of generated embryos to develop. Therefore, designed studies with larger numbers of patients receiving HU during pregnancy, with longer follow-up of exposed children and more careful assessment of embryo/fetotoxic effects, are required before this agent can be promoted as safe in pregnancy.

## 1. Introduction

Hydroxyurea (HU) is a low molecular weight nonalkylating myelosuppressive agent that impairs DNA synthesis through the inhibition of ribonucleotide reductase [1]. This drug is often used to supplement phlebotomy for patients with polycythemia vera (PV; [2]) and to reduce significantly the incidence of recurrent thrombosis in patients with essential thrombocythemia who are at high risk [3]. Even though the therapeutic use of HU may be declining in patients with chronic myeloid leukemia [4], its use for the treatment of patients with sickle cell disease (SCD; [5]) is gaining acceptance. In the latter patients, HU alleviates the classical symptoms of the SCD not by its anti-mitotic effect but by provoking increased production of fetal hemoglobin which has a higher oxygen carrying capacity than sickle cell hemoglobin. Currently, HU is used investigatively for the treatment of human immunodeficiency virus (HIV) infection [6,7]. In the latter treatment, HU does not have a direct anti-virus activity; rather, it inhibits the cellular enzyme ribonucleotide reductase and as a consequence, reduces deoxynucleotide triphosphates (dNTPs) that are necessary for DNA synthesis. Depletion of the dNTP pool results in the arrest of the cell cycle at the G1 phase prior to DNA synthesis in HIV-infected cell. Incomplete reverse transcription of the viral genome also results from the depletion of dNTP pool [6].

Although HU is an approved drug for the treatment of the above mentioned diseases by the Food and Drug Administration, it poses a risk to the developmental competence of preimplantation embryos because of its anti-mitotic effect. Some teratogenic effects of HU have been observed in fetuses of treated pregnant rodents [8–10], hence the advice to women not to attempt pregnancy while on HU. However, several case reports suggest that HU may have minimal or no teratogenic effects on *in vivo* exposed developing human fetuses. Fourteen cases of hydroxyurea therapy in pregnant patients with acute or chronic myelogenous leukemia, primary thrombocythemia, or SCD have been reported [11]. Three pregnancies were terminated by elective abortion; 1 woman developed eclampsia and delivered a phenotypically normal stillborn infant. All other patients delivered live, healthy infants without congenital anomalies. The common denominator among the human studies is that they are case studies with limited number of subjects that are not amenable to statistical scrutiny. Besides, it is not known whether the patients took HU as prescribed or when pregnancy ensued. Because HU has antiproliferative properties due to its ability to inhibit DNA synthesis, we believe that it can hinder the active proliferation and growth of preimplantation embryos. The objective of this study was to assess the risks associated with a low clinically relevant dose of HU (30 mg/kg body weight/day; [12]) used for the treatment of SCD, on ovarian function and embryo development, using C57BL/6J strain of female mice as a model.

## 2. Materials and Methods

### Animals

Adult male and female mice (strain C57BL/6J) 6–8 weeks of age were purchased from Jackson Laboratory (Bar Harbor, ME) and housed by sex (1 male or 4 females/cage) in stainless steel cages and allowed to acclimatize to the Animal Care Facilities for one week prior to initiation of studies. Mice were maintained in an environmentally controlled room with a 14-hour light and 10-hour dark cycle (lights on at 0600h), 22 °C and a humidity range of 50–60%. All animals were allowed ad libitum access to commercial mouse chow and water.

#### Experiment 1

Female mice were randomly assigned to a treatment or a control group (N = 20/group). Treatment consisted of 30 mg HU/kg administered daily by oral gavage for 28 days. The dose of HU used in this study is similar to the daily dose of this drug routinely administered to sickle cell patients [12]. Control mice were administered with the vehicle to this drug (saline) as described above. Five days prior to the cessation of treatments, folliculogenesis was induced in each animal with an IP injection of 5 IU of pregnant mare serum gonadotropin (PMSG) between 1,300 and 1,400 h. Forty eight hours post PMSG, 5 mice/group were anesthetized with isoflurane following which, laparotomy was performed to permit blood collection via the inferior vena cava puncture into serum separation tubes. Sera were separated from blood cells by centrifugation at 1,500 × g for 10 minutes and stored frozen at −20°C until assayed for estradiol-17β (E_2_).

### Ovulation Induction, Embryo Recovery and in vitro Culture

**Figure 1.**
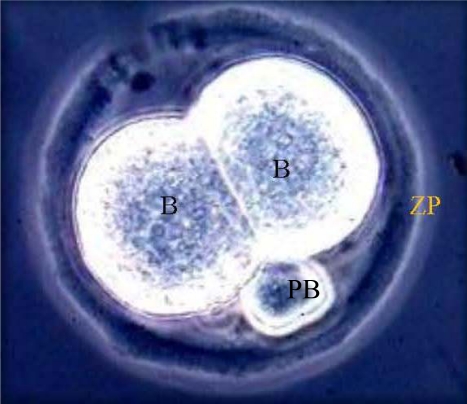
Normal 2-cell embryo with two equal blastomeres (B), one of two polar bodies (PB) encompassed by an intact zona pellucida (ZP; magnification = 400X).

Ovulation was induced at approximately 48 hours post PMSG in each of the remaining treated and control mice with an IP injection of 5 IU of human chorionic gonadotropin (hCG). Induced mice were subsequently placed with proven breeder males in a 1:1 ratio. The following morning, female mice were inspected for the presence of vaginal plugs as evidence of mating. Five mated females in the HU treatment or control group were sacrificed by CO_2_ asphyxiation immediately post detection of vaginal plugs (approximately 14–16 h post hCG), oviducts excised and flushed with Whitten’s medium (WM; [13]) containing 0.1% hyaluronidase and each flushing examined with a Nikon TMD inverted microscope at 200× magnification for the presence of cumulus masses. Each oviductal flushing was incubated at room temperature for approximately 10 minutes to permit the dispersion of cumulus cells from ovulated ova (one-cell embryos or non-fertilized oocytes). An ovum was considered fertilized if it contained 2 pronuclei in their cytoplasm and 2 polar bodies in the perivitelline space. Fertilized and non-fertilized ova were separated from the cumulus cells using pulled pipettes with the aid of an Olympus dissecting microscope. The total number of fertilized and non-fertilized oocytes per mouse was counted and considered as ovulation rate. The remaining mated mice were sacrificed at 26 to 28 hours after detection of vaginal plugs, ovaries excised and dissected free of fat tissue and weighed. Oviducts were removed and flushed with WM [13] lacking bovine serum albumin (BSA). All procedures involving animal care, anesthesia, euthanasia, and tissue collection were approved by the Meharry Medical College Institutional Animal Use and Care Committee. The flushings were examined with a Nikon TMD inverted microscope at 200× magnification for the presence of 2-cell embryos. Figure 1 depicts a photomicrograph of a normal 2-cell mouse embryo. Normal 2-cell mouse embryos, each defined as an embryo with two blastomeres of equal size with two polar bodies in the perivitelline space and encompassed by an intact zona pellucida were pooled among mice within treatment and washed in several droplets of WM containing 1% BSA (Sigma Chemical Co., St. Louis, MO).

Embryos were cultured in WM supplemented with CZBt medium containing glucose and growth factors (glutamine and taurine) and 0.3% BSA (CM) at 37ºC in an atmosphere of 5% CO_2_ in air, for five days.

### Radioimmunoassay

Serum samples were analyzed for E_2_, using radioimmunoassay previously validated in our laboratory [14]. The sensitivity of E_2_ assay was 2 pg/tube and the intra-and inter-assay coefficients of variation were 4.9 and 10.8%, respectively.

#### Experiment 2

##### In vitro *Culture of Embryos in Bioavailable HU*

Folliculogenesis and ovulation were induced in adult female C57BL/6J strain of mice (N = 10) and mated to breeder males as described in experiment 1. Two-cell embryos were recovered at the time line indicated in experiment 1 and washed as detailed in experiment 1. Normal 2-cell embryos were selected based on the criteria listed in experiment 1 and subjected to continuous *in vitro* culture in CM in the presence or absence of bioavailable HU (steady serum concentration post HU treatment = 18.0 μg HU/mL for an hour before significant reduction close to nadir; [15]) at 37 ºC in an atmosphere of 5% CO_2_ in air, for five days. At the end of the culture period, the percentage of embryos at blastocyst stage of development in the HU-treated and control group was evaluated.

#### Experiment 3

##### *Intermittent Culture* in vitro, *of Embryos in Bioavailable HU*

Experiment 3 was essentially a repetition of experiment 2 except that 2-cell embryos in the treatment group were cultured in CM containing HU (18.0 μg HU/mL) for an hour followed by two washes in CM prior to *in vitro* culture in the absence of HU. This experiment was designed to simulate the *in vivo* exposure of embryos to bioavailable HU. Embryos in the control group were exposed to saline as described for embryos in the HU treatment group prior to being cultured in CM. This process was repeated everyday for the 5 days of *in vitro* culture. At the end of culture period, the percentage of embryos at blastocyst stage of development in the intermittent HU-treated and control group was evaluated.

### Statistical Analyses

Data on ovarian weight, ovulation rate and serum concentrations of E_2_ were compared by unpaired “t” test while those on fertilization rate and embryo development were analyzed by Chi-Square.

## 3. Results

**Figure 2.**
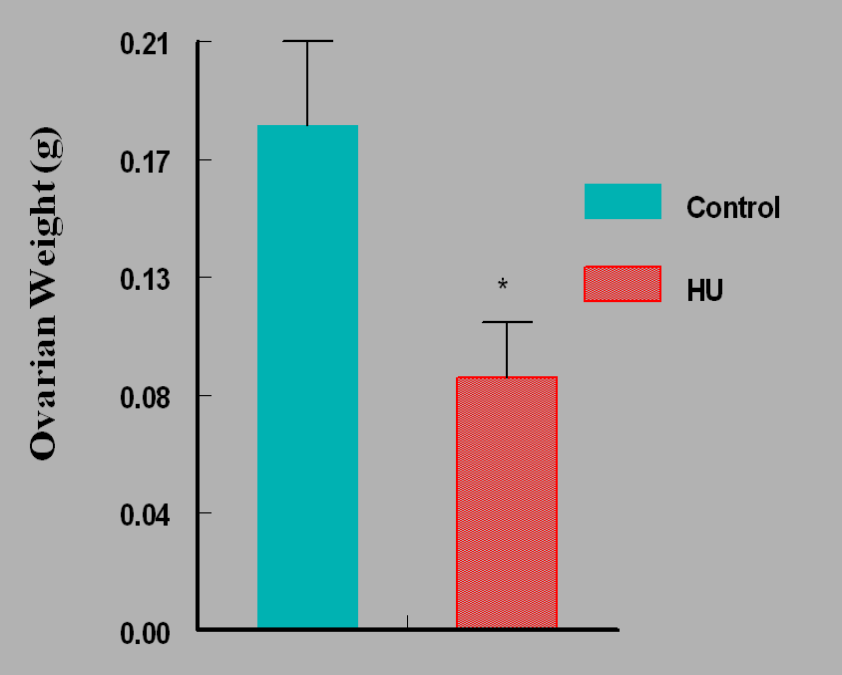
Mean Ovarian Weight of HU-treated *Versus* Control Mice.

**Figure 3.**
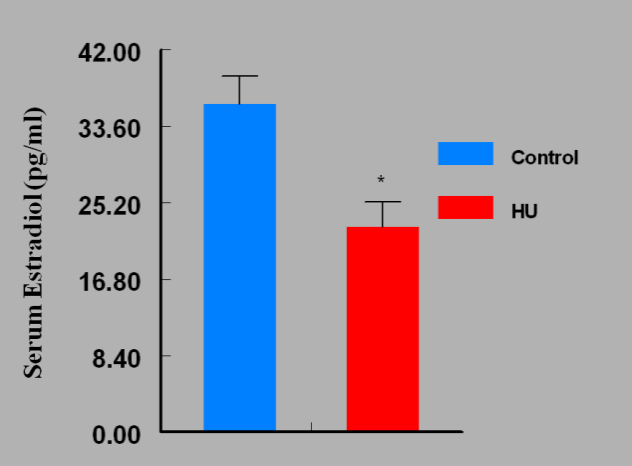
Effect of HU on Serum E_2_ Concentrations in Female Mice Treated IP with 30 mg HU/kg for 28 days; n = 5 per Group. Results are expressed as mean ± SE (HU = treated mice; Control = vehicle treated mice. Asterisks indicate a significant difference from controls (*P* < 0.05).

**Figure 4.**
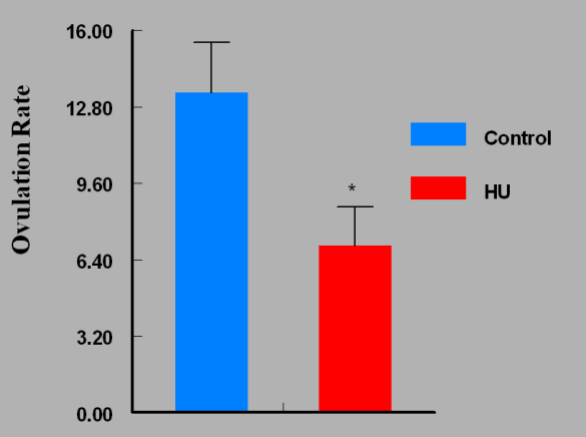
Effect of HU on Ovulation Rate in Female Mice Treated IP with 30 mg HU/kg for 28 days; n = 5 per Group. Results are expressed as mean ± SE (HU = treated mice; Control = vehicle treated mice. Asterisks indicate a significant difference from controls (*P* < 0.05).

Mice in the HU treatment group sustained approximately 50% reduction (P < 0.05) in ovarian weight compared with their counterparts in the control group (Figure 2). Interestingly, the reduction in mean ovarian weight among HU treated mice was accompanied by concomitant reductions in serum E_2_ concentrations (P < 0.05; Figure 3) and ovulation rate (P < 0.05; Figure 4).

**Figure 5.**
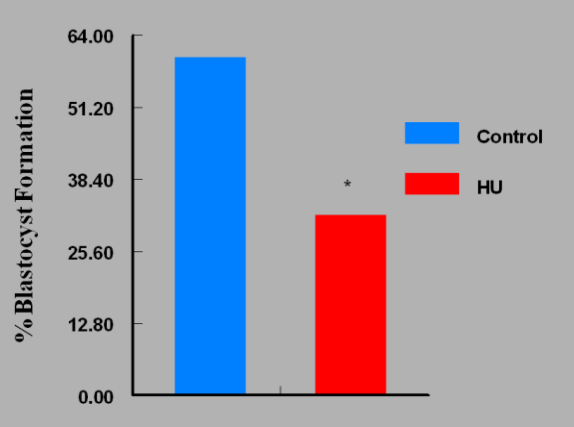
Effect of *in vivo* Exposure of Embryos to HU on Their Ability to Develop to Blastocyst Stage; n = 60 per Group. Results are expressed in percentages (HU = treated mice; Control = vehicle treated mice. Asterisks indicate a significant difference from controls (*P* < 0.05).

**Figure 6.**
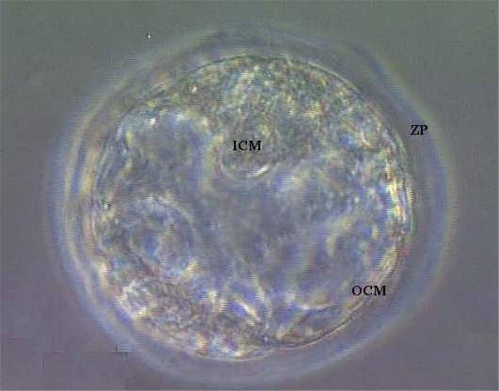
Photomicrograph of a blastocyst with an outer cell mass (OCM), inner cell mass (ICM) encompassed by an intact zona pellucida (ZP; Magnification = 400X).

Fertilization rate of oocytes recovered from HU-treated mice was 93%, comparable with that of recovered oocytes from control mice (95%). The ability of apparently normal 2-cell embryos recovered from HU-treated mice to progress to 4-cell stage of development in 36h of *in vitro* culture was comparable with their counterparts recovered from control mice. However, *in vitro* culture of *in vivo* generated 2-cell embryos (60 embryos/group) revealed that fewer (P < 0.05) *in vivo* HU-exposed embryos developed to blastocyst stage (19 [32%]); Figure 5 compared with 36 (60%) of their control counterparts that progressed to blastocyst stage of development *in vitro* (Figure 6). Of the remaining embryos, more embryos were arrested at the 8-cell stage of development among *in vivo* HU-exposed (73%; P < 0.05) compared with controls (29%) during the 5 day *in vitro* culture.

**Figure 7.**
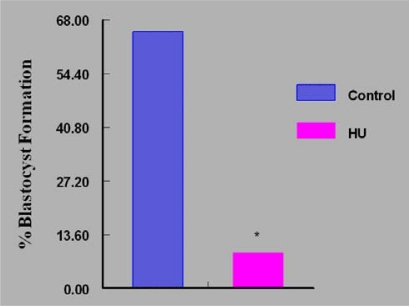
Effect of Continuous *in vitro* Exposure of Embryos to Bioavailable HU (18 μg/mL) on Their Ability to Develop to Blastocyst Stage; n = 50 per Group. Results are expressed in percentages (HU = treated embryos; Control = vehicle treated embryos. Asterisks indicate a significant difference from controls (*P* < 0.05).

**Figure 8.**
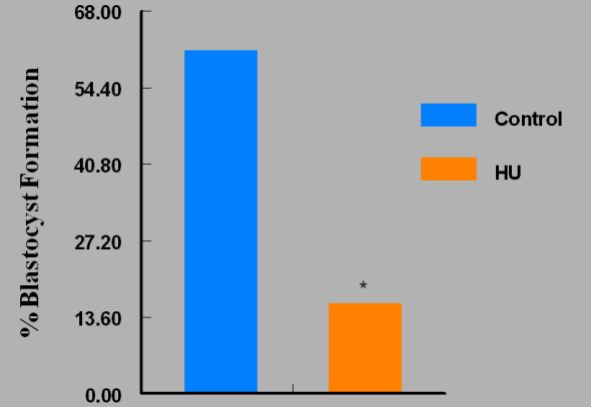
Effect of Intermittent *in vitro* Exposure of Embryos to Bioavailable HU (18 μg/mL) on Their Ability to Develop to Blastocyst Stage; n = 60 per Group. Results are expressed in percentages (HU = treated embryos; Control = vehicle treated embryos. Asterisks indicate a significant difference from controls (*P* < 0.05).

On the contrary, fewer (P < 0.05) *in vivo* HU-exposed embryos were arrested at the morulla stage (27%) compared to their control counterparts (71%) during *in vitro* culture. The continuous culture of embryos in bioavailable concentration of HU *in vitro* resulted in approximately 88% reduction in the number of embryos that attained the blastocyst stage of development compared with controls (Figure 7). However, the intermittent exposure of embryos to bioavailable concentration of HU offered a modest improvement in the percentage of embryos that attained the blastocyst stage of development (Figure 8). This regimen of embryo culture resulted in a 75% reduction in the number of embryos that attained blastocyst stage compared with their control counterparts.

## 4. Discussion

In Experiment 1, we wanted to determine if HU treatment of adult female mice compromised the ability of the *in vivo* exposed ovary to function maximally. In this experiment, we observed that ovarian weight, the key predictor of normal folliculogenesis (E_2_ production) and ovulation rate were significantly reduced in HU-treated adult mice compared with their control counterparts, indicating hypogonadism among HU-treated mice. We have shown that HU is an endocrine disruptive chemical [12] and could have very specific effects on the different tissues within the ovary. Because of the intense interdependency of the different tissues within the ovary, perturbation of the function of one or all the tissues in the ovary by HU can compromise normal folliculogenesis, ovulatory process, fertilization of ovulated ova and subsequent embryo development.

During each ovarian cycle, follicular development beyond the early antral stages is absolutely dependent upon FSH [16] for both proliferation and differentiation of granulosa cells and the synthesis of E_2_ via aromatization of androgen from the thecal tissue [17]. The resulting secondary follicles are them recruited for possible selection as dominant follicles that are triggered to ovulate under the stimulatory influence of E_2_-regulated LH surge. It is likely that the reduction in ovulation rate observed in this study among HU-treated *versus* control mice resulted from the inhibition of granulosa cell ribonucleotide reductase, resulting in the depletion of deoxyribonucleotide pools and subsequently arresting granulosa cell proliferation at late G1/early S-phase [18]. Furthermore, HU-induced reduction in DNA synthesis [1] can induce granulosa cell death among HU-treated *versus* controls. Such actions of HU on granulosa cells can negatively impact FSH-induced proliferation and differentiation of these cells and consequently lead to reduced numbers of follicles recruited for possible selection as dominant follicles; hence the reduced ovulation rate among HU-treated compared with control mice.

Numerous studies have measured steroid hormones in follicular fluid as a method for distinguishing between healthy and atretic follicles [19–22]. The inference drawn from these studies is that healthy follicles produce higher concentrations of E_2_ than either progesterone or androgen, while the reverse is true for atretic follicles [19,20,22]. It is conceivable that the significant reduction in serum E_2_ concentrations at 48 hours post PMSG (proestrus) in HU-treated mice is a reflection of fewer healthy follicles progressing towards ovulation compared with their control counterparts. Hence, the reduced ovarian weight observed among HU-treated *versus* control mice is secondary to fewer corpora lutea, the most prominent tissues in the ovary immediately following ovulation, due to fewer numbers of healthy follicles that ovulated. In this study, fertilization rates of ova recovered from HU-treated and control mice were comparable, suggesting that *in vivo* exposure of oocytes to HU did not interfere with the fertilization process.

Based on data raised in HeLa cells, HU exhibits antiproliferative effect in a dose-dependent manner [23] on proliferating cells and less so on non-proliferating cells. These are expected observations, because HU mainly affects DNA synthesis, thereby interfering with the growth of proliferating cells. Yeo *et al.* [24] found that HU inhibits the growth of human diploid fibroblasts by inducing increasing p53 and p21 levels in human diploid fibroblast cells.

In this study, mice were treated with 30 mg HU/kg once every 24 hours for 28 days. With a half-life of 2–4 hours, it is possible that the daily bioavailable levels of HU reaching the granulosa cells of maturing follicles were not significant enough to totally inhibit proliferation. Consequently, enough granulosa cells were spared to prepare oocytes to respond to hCG for the attainment of the final meiotic maturation stage (MII) and ovulate ova equipped to facilitate normal fertilization. The percentage of embryos generated towards the tail end of HU treatment that attained morulla or blastocyst stage of development was significantly reduced during *in vitro* culture in the absence of HU compared with their control counterparts. The ability of some *in vivo* HU-exposed embryos to develop to blastocyst stage indicates resilience to this therapeutic agent by exposed embryos. Embryos being diploid cells are vulnerable to the antiprolifarative action of HU [24]. If this premise is true, none of the embryos expose *in vivo* to HU would develop to blastocyst stage. Perhaps the duration of exposure of *in vivo* generated embryos to this therapeutic agent was too short to completely arrest development. Furthermore, the removal of the embryos from *in vivo* exposure and placement in an *in vitro* culture medium without further exposure to HU may have reversed any arrest imposed by HU. According to Linke *et al.* [18] HU-induced depletion of deoxyribonucleotide pools and the consequent cell cycle arrest at late G1/early S-phase is reversible. The other scenario is that the short duration of *in vivo* exposure of 2-cell embryos to HU may have resulted in uneven arrest of the blastomeres. Because blastomeres at the early cleavage stage are totepotent, one viable blastomere can rescue the entire embryo that can develop to blastocyst stage in the absence of HU during *in vitro* culture.

Experiment 2 and 3 were designed to determine if exposure of 2-cell embryos, not previously exposed to *in vivo* HU, but to continuous or intermittent HU *in vitro* affected development. The continuous *in vitro* culture of embryos in the presence of HU caused a significant percentage of embryo death due to irreversible arrest of blastomere proliferation and subsequent apoptotic degeneration [18,23]. Intermittent cultures of embryos in the presence of HU slightly reduced the percentage of dead embryos compared with those cultured continuously. This is likely due to arrests imposed on the embryos in culture by HU and arrest reversal post wash followed by a 23 hour culture before re-exposure. This regimen of *in vitro* embryo culture led to only a few more embryos progressing to blastocyst stage of development compared with their counterparts in the continuous regimen of embryo culture in the presence of HU.

Although HU is a potential antineoplatic agent, inhibitor of viral development, a beneficial drug for the alleviation of clinical manifestations of SCD and a well tolerated drug, it is highly toxic to preimplantation embryos. The ability of some embryos exposed to HU to develop to blastocyst stage indicates resilience to xenobiotics by some embryos. However, subtle damage may still occur in the surviving embryos and may be expressed post partum if exposure is high enough and continuous. Patients on HU that are considering a family should consult with their physicians before they attempt pregnancy.
